# Decreased attenuation difference between non-contrast and portal-venous phases of CT predicts the ultrasonography-unspecified adnexal torsion

**DOI:** 10.1186/s13244-024-01885-4

**Published:** 2025-01-10

**Authors:** Weili Xie, Zhongren Huang, Hongmei Kuang, Xiaoxing Li, Rixin Zhang, Wei Zeng, Cheng Jin, Junyuan Zhong, Jidong Peng, Weiling Cheng, Fuqing Zhou

**Affiliations:** 1 Department of Radiology, The First Affiliated Hospital, Jiangxi Medical College, Nanchang University, Nanchang, China; 2 Clinical Research Center for Medical Imaging in Jiangxi Province, Nanchang, China; 3 Jiangxi Province Medical Imaging Research Institute, Nanchang, China; 4 Medical Imaging Center, Ganzhou People’s Hospital, The Affiliated Ganzhou Hospital of Nanchang University and Southern Medical University, Ganzhou, China

**Keywords:** Ovarian torsion, Diagnostic imaging, CT, Acute abdomen, Emergency medicine

## Abstract

**Objectives:**

To evaluate the value of contrast-enhanced CT in diagnosing ultrasonography-unspecified adnexal torsion (AT).

**Methods:**

Surgically confirmed patients with painful pelvic masses (*n* = 165) were retrospectively collected from two institutes. Two senior radiologists independently reviewed the CT images and determined the Hounsfield unit difference between non-contrast vs portal venous phases (ΔHU_PV-NC_) in both derivation and validation samples. The cutoff value, sensitivity, specificity, predictivity, and reproducibility of the ΔHU_PV-NC_ and other visually assessed CT signs were analyzed and compared using the receiver-operating characteristic curve, multivariable regression, and inter-rater agreement assays, respectively.

**Results:**

Women with twisted (*n* = 73 [47 ± 19 years]) or untwisted (*n* = 92 [40 ± 15 years]) adnexal lesions were reviewed. The ΔHU_PV-NC_ ≤ 17.5 HU (AUC: 0.91 [95% CI: 0.86, 0.96]; sensitivity: 95% [95% CI: 87, 98]; and specificity: 88% [95% CI: 80, 94]) was the independent predictor of AT (OR: 137 [95% CI: 39, 481], *p* < 0.001). After training in ΔHU_PV-NC_ measurement, the agreement between two junior residents and the consensus increased from fair (resident-1: 0.29 [95% CI: 0.17, 0.41]; resident-2: 0.24 [95% CI: 0.1, 0.39]) to substantial (resident-1: 0.75 [95% CI: 0.65, 0.85]; resident-2: 0.72 [95% CI: 0.62, 0.83]). The post-training diagnostic accuracy (both residents: 81% [95% CI: 74, 87]) was higher than the pre-training accuracy (resident-1: 67% [95% CI: 59, 74], *p* = 0.007; resident-2: 66% [95% CI: 58, 73], *p* = 0.002).

**Conclusion:**

The sign of ΔHU_PV-NC_ ≤ 17.5 HU in contrast-enhanced CT can be used to predict the ultrasonography-unspecified AT.

**Critical relevance statement:**

The decreased attenuation difference between non-contrast vs portal venous phases, a quantitative measurement-based CT sign, highlights the value of using contrast-enhanced CT as a second-line imaging approach after an equivocal ultrasonographic examination to diagnose AT in emergency settings.

**Key Points:**

- The value of contrast-enhanced CT in diagnosing ultrasonography-unspecified AT is underestimated.
- The ΔHU_PV-NC_ ≤ 17.5 HU is the only predictor to diagnose the ultrasonography-unspecified AT.
- Contrast-enhanced CT can be used as a second-line imaging approach after an equivocal ultrasonographic examination.

**Graphical Abstract:**

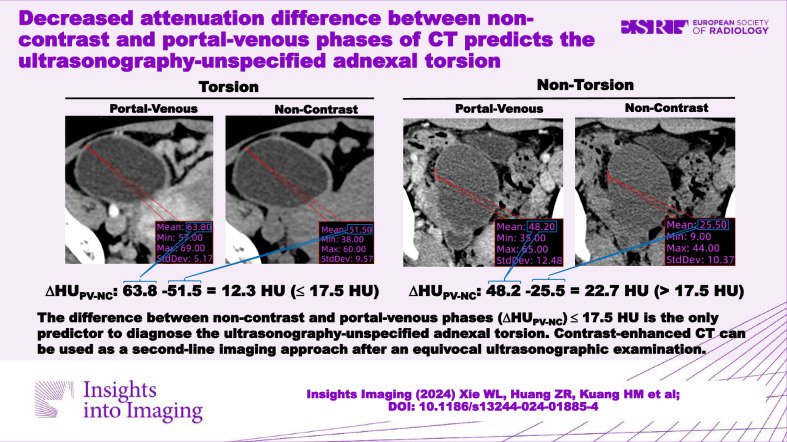

## Introduction

Adnexal torsion (AT) is a gynecological emergency characterized by the ovary and/or fallopian tube rotation around the vascular and ligamentous supports [1, 2]. Delayed diagnosis and treatment will lead to ovary necrosis and potentially long-term reduction of fertility [1, 2]. More severely, the necrosed tissue can release cytokines causing fatal complications such as peritonitis and thrombophlebitis [3]. While the clinical presentation of AT may include pelvic pain, nausea, vomiting, fever, and tenderness on palpation, the symptoms and signs are often non-specific [1, 2]. Medical imaging, such as ultrasonography, CT, and MRI have been used to diagnose AT. Still, the accuracy of preoperative diagnosis remains to be improved [4].

Ultrasonography is frequently the first imaging choice for suspected patients due to its safety, availability, and affordability [5]. The typical ultrasonographic signs include adnexal mass, whirlpool sign, ovarian edema, and pelvic fluid with pooled sensitivity and specificity varying from 53% to 69% and 46% to 95%, respectively [6]. The imbalance between the low sensitivity and high specificity makes it difficult to distinguish AT in patients lacking highly specific signs, such as the whirlpool sign. Therefore, imaging signs with high sensitivity and uncompromised specificity are urgently needed to improve the diagnostic accuracy of AT further.

While MRI shows added value in diagnosing AT in patients with uncertain ultrasonographic impressions [7], the longer scanning time, limited availability, and high expenses restrict the use of this approach in an emergency setting. With advantages including short examination times, high spatial and contrast resolution, considerable image reconstruction capability, and good-to-excellent inter-observer reliability, CT scanning in the emergency department has increased by more than threefold during the past decades, especially for patients with acute abdominal pain [8, 9]. While the proper use of CT in emergency settings largely changes the physician’s decision-making and reduces diagnostic uncertainty [10], it remains to determine the value of CT in diagnosing AT, particularly in patients without a confident ultrasonographic impression [1, 5].

Since the contrast medium can quickly distribute to the capillary bed and tissue interstitial space [11, 12], contrast-enhanced CT has been used to diagnose acute ischemic diseases [13–15]. Given that the key pathophysiological characteristic of AT is the obstruction of the blood supply [1, 2], we aimed to determine whether the Hounsfield unit (HU) difference between non-contrast and portal-venous phases (ΔHU_PV-NC_) can be used to diagnose AT in patients with uncertain ultrasonographic impressions.

## Methods

### Study design and participants

The ethics committee of our institutes approved this retrospective study (file no. IIT2024082) and waived the informed consent.

The patient information was consecutively retrieved from the database of the departments of obstetrics and gynecology at First Affiliated Hospital of Nanchang University (Institute-1) and Ganzhou People’s Hospital (Institute-2).

**Fig. 1:**
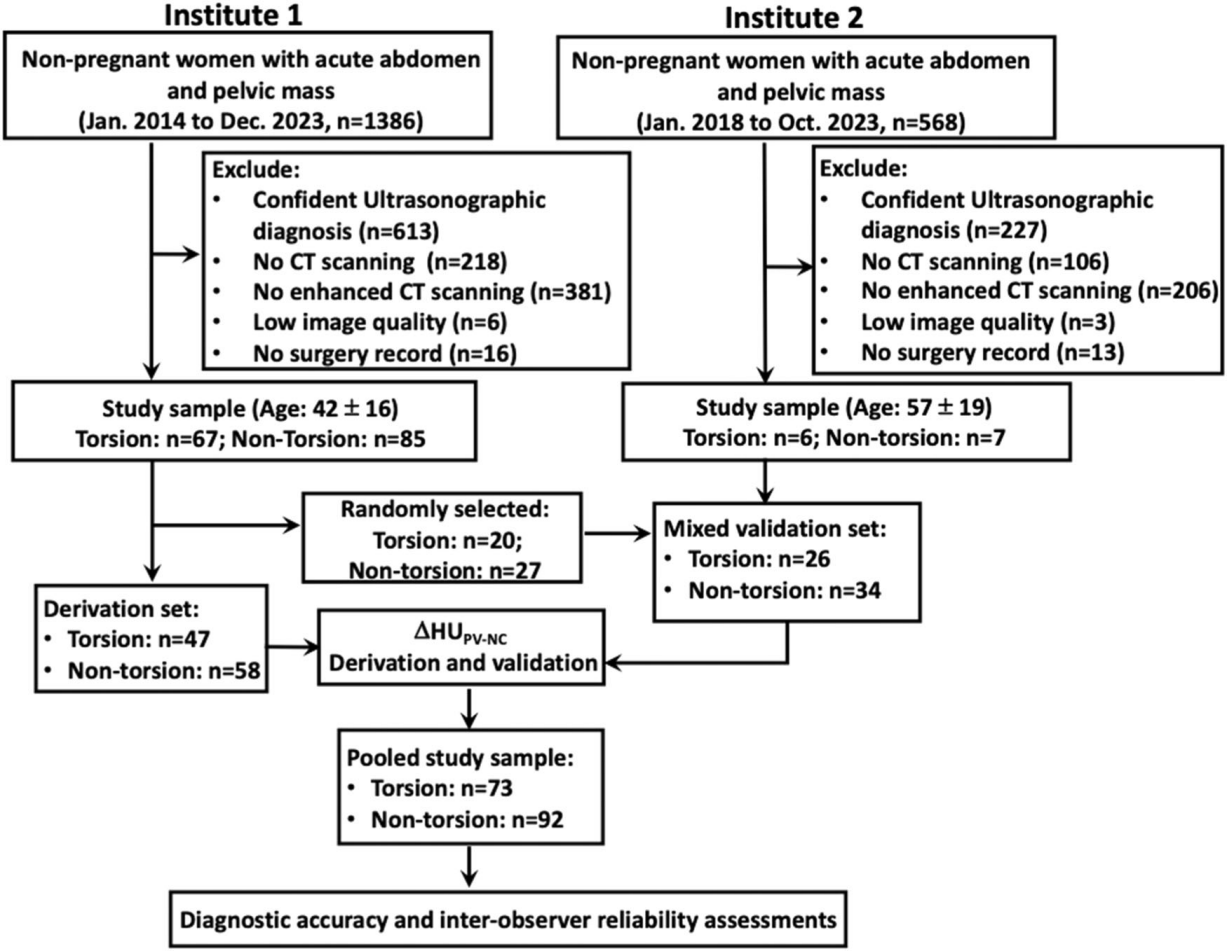
Flow chart of study design

The starting population comprised non-pregnant women with a chief complaint of acute abdominal pain and ultrasonography-confirmed pelvic mass from both Institute-1 (*n* = 1386, Jan 2014 to Dec 2023) and Institute-2 (*n* = 568, Jan 2018 to Oct 2023). The ruled-out criteria were as follows: (a) confident ultrasonographic impression, (b) no CT scanning, (c) no contrast-enhanced CT scanning, (d), low image quality, and (e) no surgery record. The study sample sizes were 152 (age: 42 ± 16 years) and 13 (age: 57 ± 19 years) for Institutes 1 and 2, respectively (Fig. 1).

Both derivation (105 patients from Institute-1) and mixed validation (13 patients from Institute-2 plus 47 patients from Institute-1) sets were established and the number of patients with or without torsion was 47 vs 58 and 26 vs 34, respectively. After ΔHU_PV-NC_ derivation and validation, the patients were combined to form a pooled study sample (torsion: 73; non-torsion: 92) to assess the diagnostic accuracy and inter-observer reliability (Fig. 1 and Table 1).

**Table 1:** Patient characteristics

	Torsion (*n* = 73)	Non-torsion (*n* = 92)	*p* value^c^
Age (y)^a^	47 ± 19	40 ± 15	0.014^d^
Pain duration (d)^a^	6 ± 6	10 ± 9	< 0.001^e^
Nausea or vomiting^b^	12 (16)	6 (7)	0.08
Neutrophil lymphocyte ratio	6.9 ± 6	4.4 ± 3.8	< 0.001^e^
Largest diameter of the lesion (cm)^a^	11.4 ± 4.5	11.1 ± 6.1	0.07^e^
Interval between CT and surgery (d)	3 ± 2	4 ± 3	0.1^e^
Pathology^b^			
Ovarian Cyst	23 (32)	22 (24)	0.29
Serous cystadenoma	13 (18)	7 (8)	0.06
Mucinous cystadenoma	9 (12)	13 (14)	0.82
Seromucinous cystadenoma	1 (1)	0 (0)	0.44
Teratoma	11 (15)	10 (11)	0.65
Fibrothecoma	3 (4)	1 (1)	0.32
Endometrioma	2 (3)	14 (15)	0.007
Fallopian tube	3 (4)	0 (0)	0.09
Polycystic ovary syndrome	1 (1)	0 (0)	0.44
Tubo-ovarian abscess	1 (1)	18 (20)	< 0.001
Fibroma	1 (1)	0 (0)	0.44
Dysgerminoma	1 (1)	0 (0)	0.44
Thecoma	1 (1)	1 (1)	1
Paramesonephric duct cyst	1 (1)	0 (0)	0.44
Mucinous cystadenocarcinoma	1 (1)	1 (1)	1
Borderline mucinous tumor	0 (0)	3 (3)	0.25
Cortical inclusion cyst	1 (1)	0 (0)	0.44
Serous cystadenocarcinoma	0 (0)	1 (1)	1
Squamous cell carcinoma	0 (0)	1 (1)	1

^a^ Data are presented as mean ± SD

^b^ Data are presented as the number of occurrences followed by (percentage)

^c^ *p* value from Fisher’s exact test except where otherwise indicated

^d^ Student *t*-test

^e^ Mann–Whitney *U*-test

### CT protocol

Since the patients were retrospectively collected from two institutes with a time range of ten years, multiple CT scanners have been used. The technical parameters of each CT scanner are summarized in Table 2. The contrast was Visipaque^TM^ (320 mgI/mL). The injection rate and dose were 2.5–4 mL/s and 1.2–1.5 mL/kg, respectively.

**Table 2:** Technical parameters of the CT scanner

Manufacturers	Models	Slice thickness, (mm)	Reconstruction interval, (mm)	Tube voltage, (kV)	Tube current, (mAs)	Rotation time, (s)	Pitch	Scan delays used for portal venous phase, (s)
Siemens Healthineers	Somatom Force	2	2	120	100–210	0.5	0.6	60–75
Siemens Healthineers	Somatom Definition AS+	2	1.5	120	60–120	0.5	0.6	60–75
Siemens Healthineers	Emotion 16	2	2	110	30–100	0.5	1.2	60–75
GE Healthcare	Revolution	1.25	1.25	120	100–250	0.5	0.992	60–75
Philips Healthcare	Brilliance iCT	2	2	120	150–210	0.75	0.800	60–75

### CT image analysis

Two senior radiologists, W.C. (12 years of experience) and H.K. (10 years of experience), blinded to the patient’s information, independently reviewed the images and determined each CT sign. Disagreements were resolved by discussion to achieve a consensus.

**Fig. 2:**
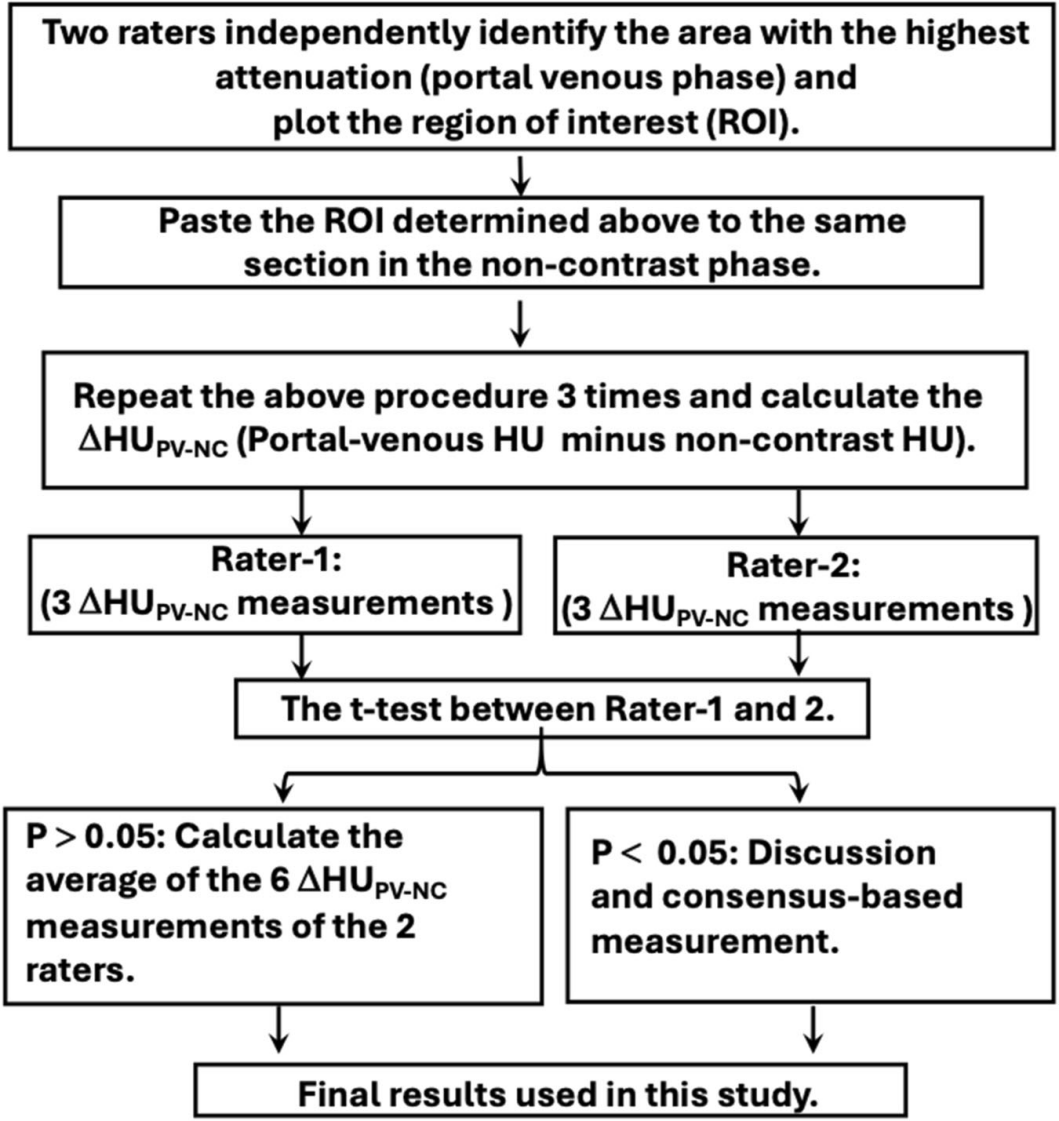
The procedure for measuring the ΔHU_PV-NC_

The HU was measured by plotting the region of interest (ROI) in the lesion according to the following stepwise procedure: (a) identify the most notably attenuated region within the lesion in the portal-venous phase, (b) draw the ROI by precisely circling the area with the highest attenuation but avoid calcification, hemorrhage, abnormal iodine contrast uptake (e.g., thyroid tissue in teratoma), and adjacent, untwisted organs (e.g., gut), (c) paste the selected region to the same section in the non-contrast image, (d) repeat the measurement for three times and calculate the ΔHU_PV-NC_ (portal-venous HU minus non-contrast HU), (e) compare the mean of the three ΔHU_PV-NC_ measured by each rater using the paired *t*-test, (f) if the *p* > 0.05, take the average of the six ΔHU_PV-NC_ as the final result, and (g) if the *p* < 0.05, discuss to achieve the consensus (Fig. 2). The visually assessed CT signs were reviewed including whirlpool sign, peritoneal ascites, lesion wall thickening, intralesional hemorrhage, and fat stranding (Table S1) [16, 17].

### Inter-observer reliability

Two medical residents, X.L. and R.Z. (two years of experience) were selected to assess the inter-observer reliability. Blinded to the patient’s clinical information and ΔHU_PV-NC_, the two residents independently reviewed all the images to make an initial diagnosis. Three months later, they were trained to calculate ΔHU_PV-NC_ using the CT images of fifteen patients not included in the study samples. A final diagnosis was made based on the post-training image review. The pre-and post-training agreement and diagnostic accuracy were assessed.

### Reference standard

The surgical and pathological reports were used as the reference standard. No adverse events were identified between the CT scanning and surgery.

### Statistical analysis

Normally distributed continuous variables were compared using the Student *t*-test. Mann–Whitney *U*-test was selected to compare variables without normal distribution. Categorical variables were presented as numbers and percentages of patients. The comparison of categorical variables was conducted using Pearson chi-square or Fisher exact test. The ΔHU_PV-NC_ was plotted in the receiver operating characteristic curve (ROC) to determine the area under the curve (AUC) and cutoff value (maximum of sensitivity + specificity). The AUC between derivation and validation ROCs was compared according to the published method [18]. The McNemar test was used to compare the sensitivity, specificity, and diagnostic accuracy [19]. Univariable logistic regression was used to assess the association between each CT sign and AT. The predictors with *p* < 0.1 were introduced in a multivariable logistic regression model followed by backward elimination to determine the independent association between the CT signs and AT. Cohen’s kappa was used to assess the inter-observer reliability between two junior residents and consensus. The kappa value was interpreted as follows: slight (0–0.20), fair (0.21–0.40), moderate (0.41–0.60), substantial (0.61–0.80), and almost perfect (0.81–1.00) [20]. SPSS-26.0 and Prism 10 were used to conduct the statistics and generate the graph. *p* < 0.05 was considered statistically significant.

## Results

### Patient’s characteristics

As shown in Table 1, the patients with AT had older age (AT: 47 ± 19 years; non-AT: 40 ± 15 years; *p* = 0.014), shorter pain duration (AT: 6 ± 6 days; non-AT: 10 ± 9 days; *p* < 0.001), and higher neutrophil-lymphocyte ratio (AT: 6.9 ± 6; non-AT: 4.4 ± 3.8; *p* < 0.001). The four most frequently seen lesion types in the AT group were ovarian cyst (32%, 23/73), serous cystadenoma (18%, 13/73), teratoma (15%, 11/73), and mucinous cystadenoma (12%, 9/73). The proportions of endometrioma and tubo-ovarian abscess were higher in the non-AT group (endometrioma: 15%, 14/92; tubo-ovarian abscess 20%, 18/92) than those in the AT group (endometrioma 3%, 2/73, *p* = 0.007; tubo-ovarian abscess: 1%, 1/73, *p* < 0.001).

### Derivation and validation of ΔHU_PV-NC_

**Fig. 3:**
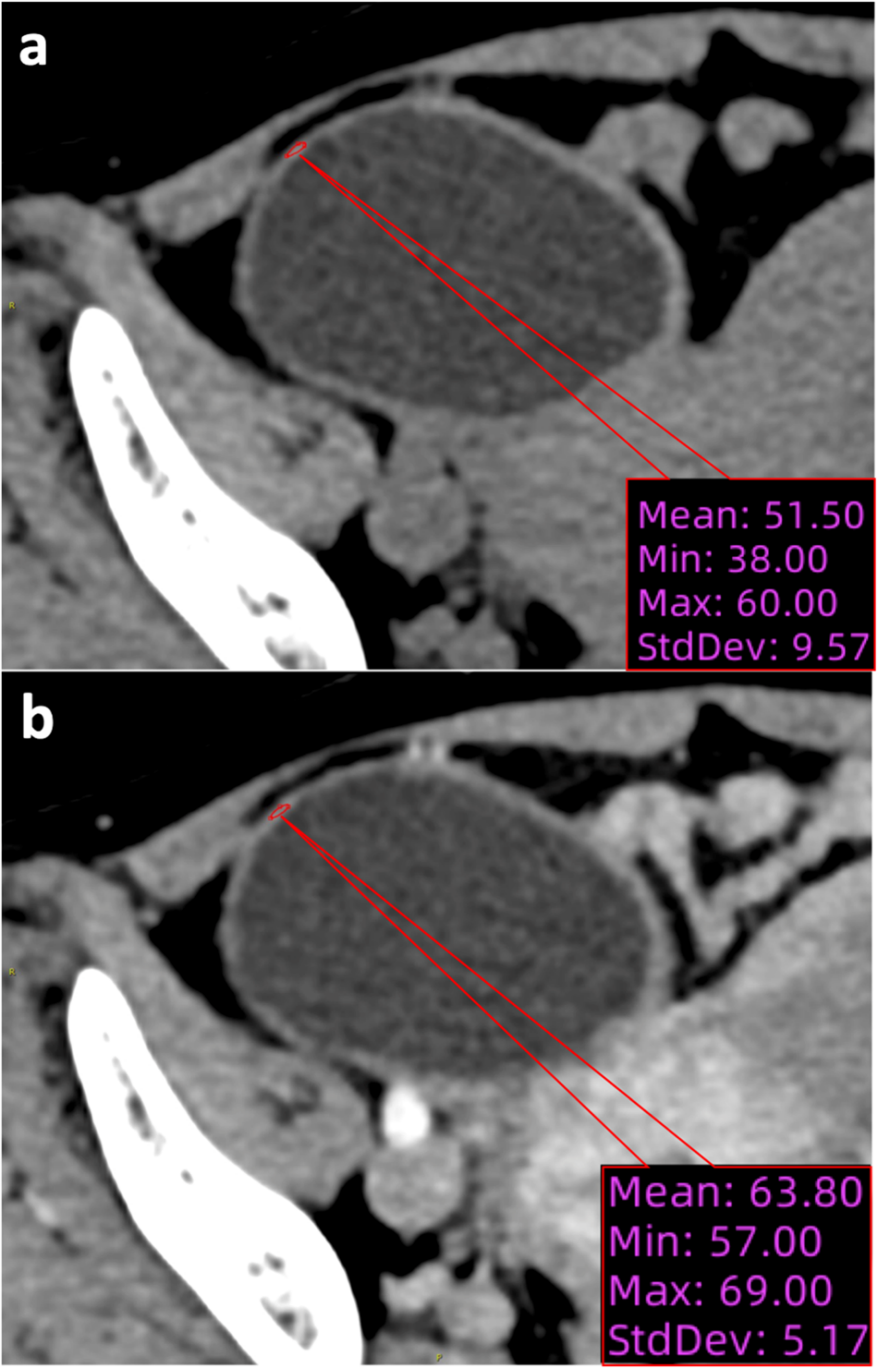
Attenuation measurement in contrast-enhanced CT of a patient (age: 53 years) with a chief complaint of abdominal pain for 1 day. Surgically and pathologically confirmed twisted ovarian cyst. **a** Non-contrast phase (attenuation: 51.5 HU). **b** Portal-venous phase (attenuation: 63.8 HU). The ΔHU_PV-NC_ is 12.3 HU

**Fig. 4:**
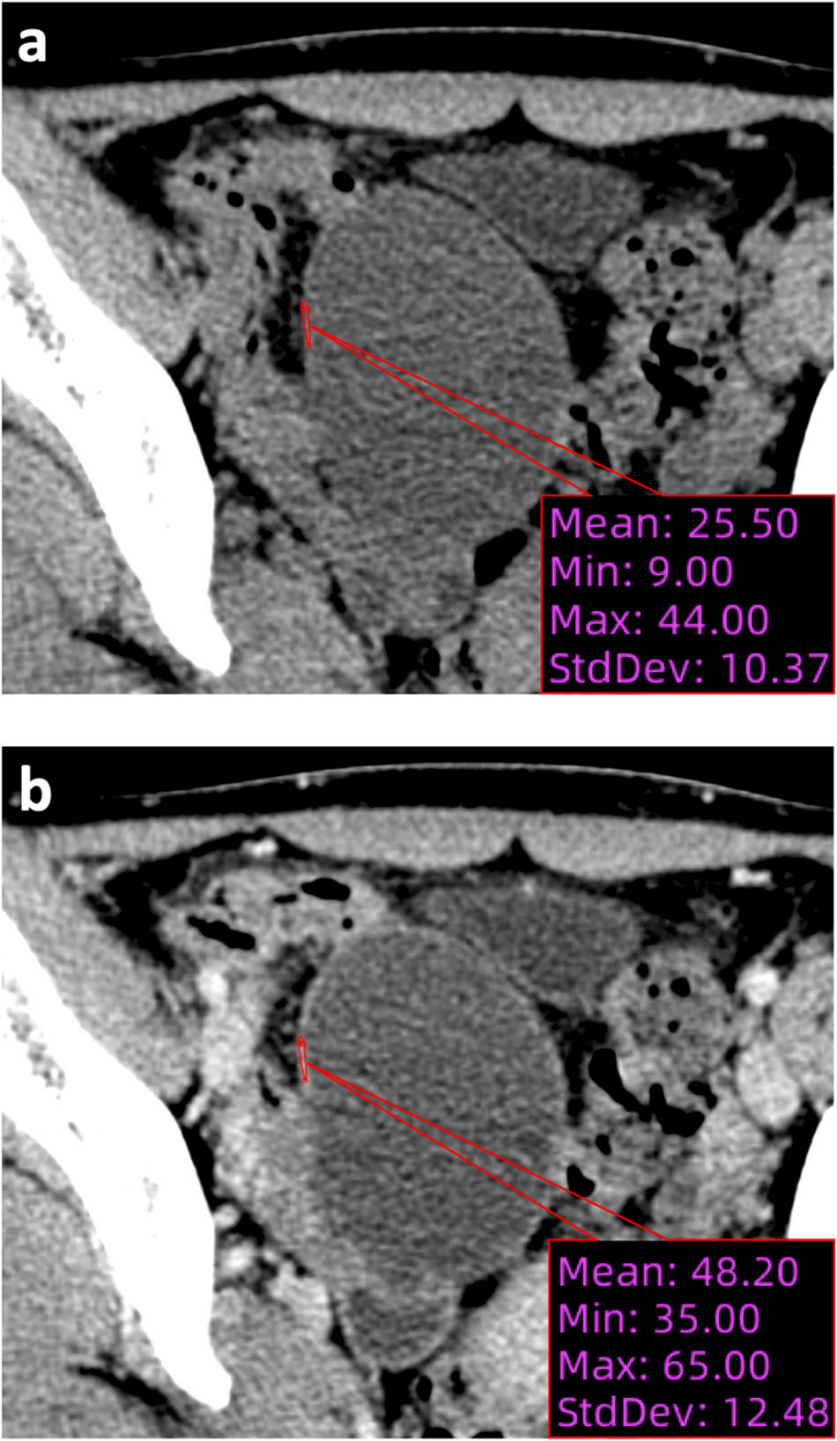
Attenuation measurement in contrast-enhanced CT of a patient (age: 22 years) with a chief complaint of abdominal pain for 4 days. Surgically and pathologically confirmed untwisted ovarian cyst. **a** Non-contrast phase (attenuation: 25.5 HU). **b** Portal-venous phase (attenuation: 48.2 HU). The ΔHU_PV-NC_ is 22.7 HU

The attenuation of the lesion was measured based on ROI plotting in contrast-enhanced CT images in both non-contrast and portal-venous phases (Figs. 3 and 4).

**Fig. 5:**
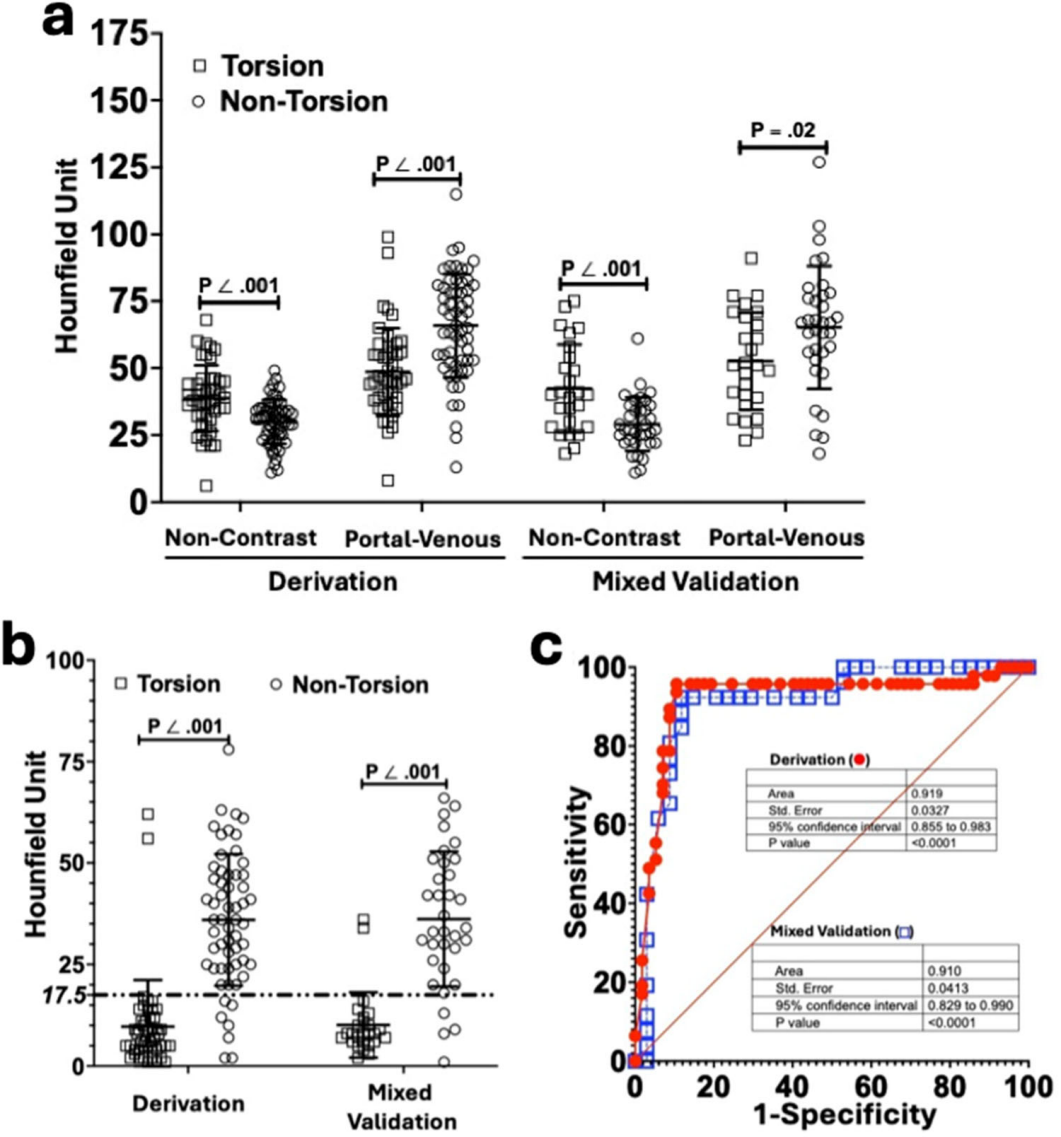
Attenuation and ΔHU_PV-NC_ in derivation and mixed validation sets. **a** Attenuation of the twisted vs untwisted lesions in derivation and mixed validation sets. Derivation set: non-contrast phase (torsion: 38.8 ± 12.2 HU; non-torsion: 29.8 ± 5.5 HU, *p* < 0.001) and portal-venous phase (torsion: 48.5 ± 16.2 HU; non-torsion: 65.6 ± 19.7 HU; *p* < 0.001). Mixed validation set: non-contrast phase (torsion: 42.5 ± 16.4 HU; non-torsion: 30 ± 9.7 HU; *p* < 0.001) and portal-venous phase (torsion: 52.6 ± 18.2 HU; non-torsion: 64.1 ± 22.6 HU; *p* = 0.02). **b** ΔHU_PV-NC_ (cutoff value: 17.5 HU) of the twisted vs untwisted lesions in derivation and mixed validation sets. Derivation set: (torsion: 9.7 ± 11.4 HU; non-torsion: 36 ± 16.2 HU, *p* < 0.001). Mixed validation set: (torsion: 10.1 ± 8 HU; non-torsion: 36.2 ± 16.6 HU, *p* < 0.001). **c** The receiver operating characteristic curve (ROC) of the ΔHU_PV-NC_ in the derivation (red solid round; torsion: *n* = 47; non-torsion: *n* = 58; sensitivity: 96% [95% CI: 86, 99]; specificity: 89% [95% CI: 79, 95]) and mixed validation (blue open square; torsion: *n* = 26; non-torsion: *n* = 34; sensitivity: 92% [95% CI: 76, 99]; specificity: 88% [95% CI: 73, 95]) sets

In the derivation set, the mean attenuation in the non-contrast phase was higher in the AT group (38.8 ± 12.2 HU) than in the non-AT group (29.8 ± 5.5 HU, *p* < 0.001, Fig. 5a). In contrast, the mean attenuation in the portal-venous phase was lower in the AT group (48.5 ± 16.2 HU) than in the non-AT group (65.6 ± 19.7 HU, *p* < 0.001, Fig. 5a). The same pattern was identified in the mixed validation set as the mean attenuation in the AT group was higher in the non-contrast phase (AT: 42.5 ± 16.4 HU; non-AT: 30 ± 9.7 HU; *p* < 0.001; Fig. 5a) but lower in the portal-venous phase (AT: 52.6 ± 18.2 HU; non-AT: 64.1 ± 22.6 HU; *p* = 0.02, Fig. 5a).

The ΔHU_PV-NC_ was measured by subtracting the non-contrast HU from portal-venous HU. In both derivation and mixed validation sets, the ΔHU_PV-NC_ in the AT group (derivation: 9.7 ± 11.4 HU; mixed validation: 10.1 ± 8 HU) was lower than those in the non-AT group (derivation: 36 ± 16.2 HU, *p* < 0.001; mixed validation: 36.2 ± 16.6 HU, *p* < 0.001; Fig. 5b). Once the ROC of ΔHU_PV-NC_ was plotted, the AUC of the derivation set (0.92 [95% CI: 0.86, 0.98]) was comparable to that of the mixed-validation set (0.91 [95% CI: 0.83, 0.99]; *p* = 0.86; Fig. 5c) [18]. With the cutoff value of 17.5 HU, the sensitivities of ΔHU_PV-NC_ in derivation (96% [95% CI: 86, 99]) and mixed-validation (92% ([95% CI: 76, 99]) sets were comparable (*p* = 0.61, Fig. 5C and Table S3). The difference in the specificity of ΔHU_PV-NC_ in derivation (89% [95% CI: 79, 95]) and mixed-validation (88% ([95% CI: 73, 95]) sets was insignificant (*p* = 1; Fig. 5c and Table S4).

**Fig. 6:**
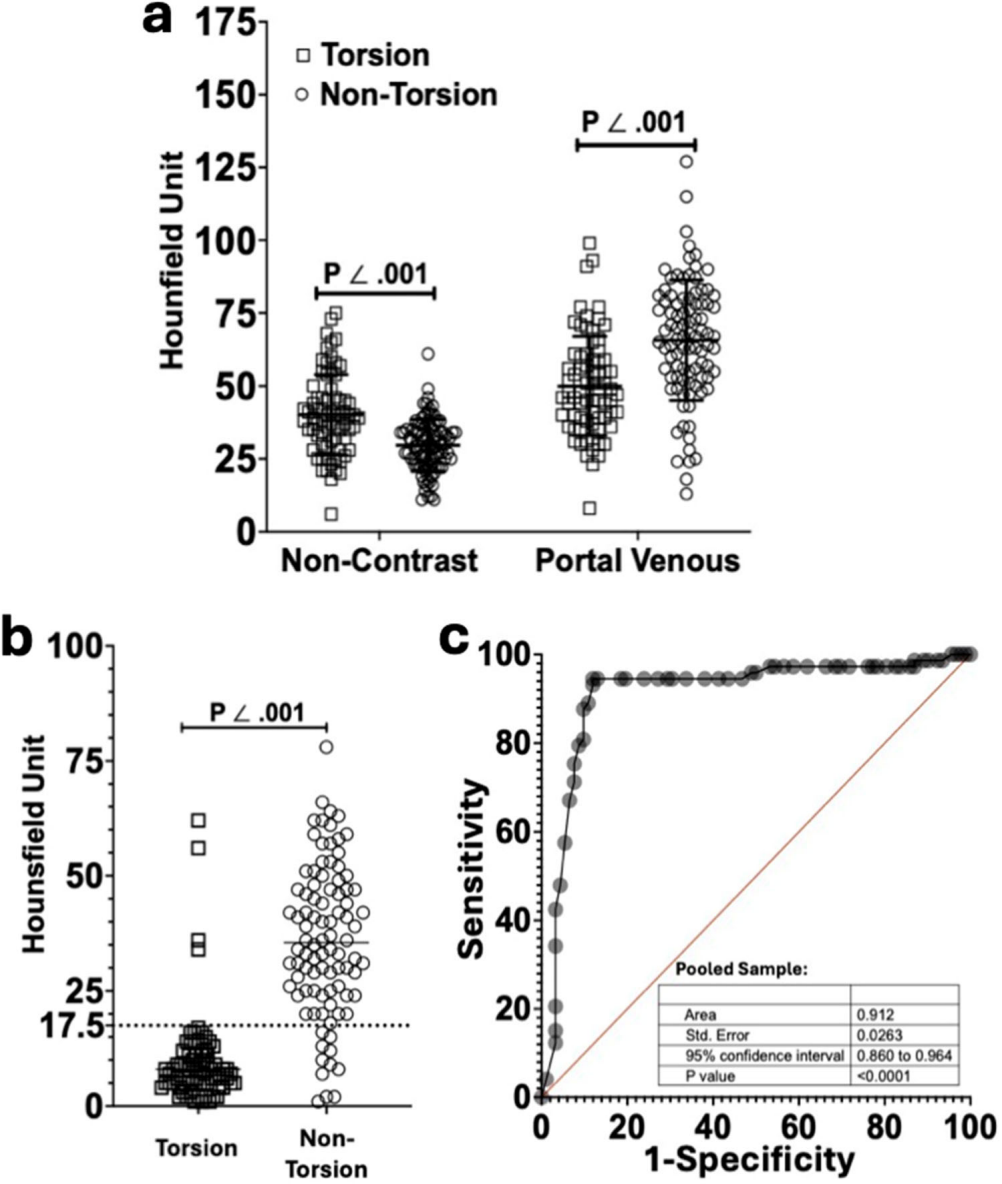
Attenuation and ΔHU_PV-NC_ in the pooled sample. **a** Attenuation of twisted vs untwisted lesions in the pooled sample. Non-contrast phase (torsion: 40.1 ± 10.7 HU; non-torsion: 29.7 ± 8.3 HU; *p* < 0.001). Portal-venous phase (torsion: 50 ± 16.9 HU; non-torsion: 68.4 ± 20.7 HU; *p* < 0.001). **b** ΔHU_PV-NC_ (cutoff value: 17.5 HU) of twisted vs untwisted lesions in the pooled sample. Torsion: 9.9 ± 10.3 HU; non-torsion: 39.3 ± 17.6 HU (*p* < 0.001). **c** The ROC of the ΔHU_PV-NC_ in the pooled sample (torsion: *n* = 73; non-torsion: *n* = 92; sensitivity: 95% [95% CI: 87, 98]; specificity: 88% [95% CI: 80, 94])

In the pooled study sample, the mean attenuation in the non-contrast phase was higher in the AT group (40.1 ± 10.7 HU) than in the non-AT group (29.7 ± 8.3 HU, *p* < 0.001, Fig. 6a). In the portal-venous phase, the mean attenuation in the AT group (50 ± 16.9 HU) was lower than in the non-AT group (68.4 ± 20.7 HU, *p* < 0.001, Fig. 6a). The ΔHU_PV-NC_ was lower in the AT group (9.9 ± 10.3 HU) than in the non-AT group (39.3 ± 17.6 HU, *p* < 0.001; Fig. 6b). The AUC, sensitivity, and specificity of the pooled sample were 0.91 (95% CI: 0.86, 0.96), 95% (95% CI: 87, 98), and 88% (95% CI: 80, 94), respectively (Fig. 6c and Table 3).

**Table 3:** Diagnostic accuracy and logistic regression of CT signs to predict AT

							Univariable regression		Multivariable regression	
CT signs	Sn^b^ (%)	*p* Value^d^	Sp^c^ (%)	*p* value ^d^	LR+^e^	LR−^f^	OR^g^	*p* value	OR^g^	*p* value
ΔHU_PV-NC_ ≤ 17.5 HU^a^	95 (87, 98)	NA^h^	88 (80, 94)	NA^h^	7.5 (4.9, 14)	0.06 (0.02, 0.16)	127 (39, 417)	< 0.001	137 (39, 481)	< 0.001
Whirlpool sign	37 (27, 49)	< 0.001	93 (87, 97)	0.17	5.7 (2.5, 13)	0.67 (0.56, 0.81)	8.4 (3.2, 21.8)	< 0.001		
Fat stranding	44 (33, 55)	< 0.001	60 (50, 70)	< 0.001	1.1 (0.8, 1.6)	0.93 (0.72, 1.21)	1.2 (0.6, 2.2)	0.005	3.4 (1, 11.8)	0.057
Peritoneal ascites	42 (32, 54)	< 0.001	71 (61, 79)	0.006	1.5 (1, 2.2)	0.81 (0.64, 1.1)	1.8 (0.9, 3.4)	0.08		
Lesion wall thickening	44 (33, 55)	< 0.001	85 (76, 91)	0.49	2.8 (1.7, 5)	0.66 (0.53, 0.83)	4.4 (2.1, 9.1)	< 0.001		
Intralesional hemorrhage	40 (29, 51)	< 0.001	77 (68, 85)	0.06	1.7 (1.1, 2.8)	0.78 (0.63, 0.97)	2.2 (1.1, 4.4)	0.02		

Values in parentheses are 95% CIs

^a^ ΔHU_PV-NC_: the HU difference between non-contrast and portal-venous phases

^b^ Sn: sensitivity

^c^ Sp: specificity

^d^ *p* value of the comparison between visually assessed CT signs and ΔHU_PV-NC_ ≤ 17.5 HU (McNemar test)

^e^ LR+: positive likelihood ratio

^f^ LR−: negative likelihood ratio

^g^ OR: diagnostic odds ratio

^h^ NA: not applicable

### Diagnostic performance of ΔHU_PV-NC_ in patients with ultrasonography-unspecified AT

The sensitivity of ΔHU_PV-NC_ ≤ 17.5 HU (95% [95% CI: 87, 98]) was higher than those of the whirlpool sign (37% [95% CI: 27, 49], *p* < 0.001), peritoneal ascites (42% [95% CI: 32, 54], *p* < 0.001), lesion wall thickening (44% [95% CI: 33, 55], *p* < 0.001), intralesional hemorrhage (40% [95% CI: 29, 51], *p* < 0.001), and fat stranding (44% [95% CI: 33, 55], *p* < 0.001; Tables 3 and S5–9). The specificity of ΔHU_PV-NC_ ≤ 17.5 HU (88% [95% CI: 80, 94]) was higher than those of peritoneal ascites (71% [95% CI: 61, 79], *p* = 0.006) and fat stranding (60% [95% CI: 50, 70], *p* < 0.001), but comparable to those of whirlpool sign (93% [95% CI: 87, 97], *p* = 0.17), lesion wall thickening (85% [95% CI: 76, 91], *p* = 0.49), and intralesional hemorrhage (77% [95% CI: 68, 85], *p* = 0.06 Tables 3 and S10–14).

Eleven and four patients were false positive and false negative for the sign of ΔHU_PV-NC_ ≤ 17.5, respectively (Table S15 and Figs. S1 and S2).

In univariable logistic regression, the AT was associated with the ΔHU_PV-NC_ ≤ 17.5 HU (OR = 127 [95% CI: 39, 417], *p* < 0.001), whirlpool sign (OR = 8.4 [95% CI: 3.2, 21.8], *p* < 0.001), lesion wall thickening (OR = 4.4 [95% CI: 2.1, 9.1], *p* < 0.001), intralesional hemorrhage (OR = 2.2 [95% CI: 1.1, 4.4], *p* = 0.02), and fat stranding (OR = 1.2 [95% CI: 0.6, 2.2], *p* = 0.005). In multivariable logistic regression, the AT was only associated with the ΔHU_PV-NC_ ≤ 17.5 HU (OR = 137 [95% CI: 39, 481], *p* < 0.001; Tables 3 and S16).

### Diagnostic performance of ΔHU_PV-NC_ in patients with ultrasound suspicion of AT

In patients with ultrasound suspicion of AT (torsion: *n* = 37, non-torsion: *n* = 16), the AUC of ΔHU_PV-NC_ was 0.94 (95% CI: 0.88, 1; Fig. S3). With the cutoff value of 17.5 HU, the sensitivity and specificity were 95% (95% CI: 82, 99) and 81% (95% CI: 54, 96; Fig. S3 and Table S17). The diagnostic accuracy of ΔHU_PV-NC_ ≤ 17.5 HU (88%, [95% CI: 75, 96]) was higher than those of whirlpool sign (68% [95% CI: 54, 80], *p* = 0.013), peritoneal ascites (53% [95% CI: 39, 67], *p* < 0.001), lesion wall thickening (66% [95% CI: 52, 78], *p* < 0.001), intralesional hemorrhage (53% [95% CI: 39, 67], *p* < 0.001), and fat stranding (57% [95% CI: 42, 70], *p* < 0.001; Table S17).

### Diagnostic performance of ΔHU_PV-NC_ in pooled patients with either ultrasonography-specified or ultrasonography-unspecified AT

Once the patients with either ultrasonography-specified or ultrasonography-unspecified AT were pooled together (torsion: *n* = 110, non-torsion: *n* = 108), the AUC of ΔHU_PV-NC_ was 0.92 (95% CI: 0.87, 0.96, *p* < 0.001; Fig. S4). With the cutoff value of 17.5 HU, the sensitivity and specificity were 95% (95% CI: 89, 98) and 87% (95% CI: 79, 92; Fig. S4), similar to the sensitivity (95% [95% CI: 87, 98], *p* = 1) and specificity (88% [95% CI: 80, 94], *p* = 1) in patients with ultrasound-unspecified AT (Tables 3, S18, and S19).

### Inter-observer reliability assessment

Before being trained with the ΔHU_PV-NC_ measurement, the agreement, assessed by Cohen’s kappa, between each resident (2 years of experience) and the consensus was 0.29 (95% CI: 0.17, 0.41) and 0.24 (95% CI: 0.1, 0.39). After the training, the inter-rater agreement increased to 0.75 (95% CI: 0.65, 0.85) and 0.72 (95% CI: 0.62, 0.83; Tables 4 and S20–S23). The pre-training diagnostic accuracy of resident-1 and resident-2 were 67% (95% CI: 59, 74) and 66% (95% CI: 58, 73), respectively. After the training, the diagnostic accuracy was improved (resident-1: 81% [95% CI: 74, 87], *p* = 0.007; resident-2: 81% [95% CI: 74, 87], *p* = 0.002; Tables 4 and S24–S29).

**Table 4:** Diagnostic accuracy and inter-observer reliability of two less-experienced residents before and after the training of ΔHU_PV-NC_^a^

	Cohen’s kappa^b^		Diagnostic accuracy		
Raters	Pre-training	Post-training	Pre-training	Post-training	*p* value^c^
Resident 1	0.29 (0.17, 0.41)	0.75 (0.65, 0.85)	67 (59, 74)	81 (74, 87)	0.007
Resident 2	0.24 (0.1, 0.39)	0.72 (0.62, 0.83)	66 (58, 73)	81 (74, 87)	0.002

Values in parentheses are 95% CIs

^a^ ΔHU_PV-NC_: HU difference between non-contrast and portal-venous phases

^b^ Agreement between each resident and the consensus between the two senior radiologists

^c^ *p* value of the comparison between pre- vs post-training diagnostic accuracy using the McNemar test

## Discussion

It is challenging to make preoperative diagnoses of AT in patients with equivocal ultrasonographic impressions. Here we show that the ΔHU_PV-NC_, with a cutoff value of 17.5 HU, has a sensitivity and specificity of 95% (95% CI: 87, 98) and 88% (95% CI: 80, 94), respectively. Being the only predictor (OR: 137 [95% CI: 39, 481], *p* < 0.001) among all the CT signs assessed in this study, the introduction of ΔHU_PV-NC_ improved the diagnostic accuracy and inter-observer agreement in the less-experienced medical residents after a brief training. Our study suggests that contrast-enhanced CT is a valuable imaging modality to diagnose AT in suspected patients with inconclusive ultrasonographic impressions.

Poor enhancement of the twisted adnexal masse has been reported in prior studies [7, 21–25], whereas the published work has deficiencies such as the absence of a disease-free control group and lack of quantitative measurements [7, 21–23, 25]. While Lee et al, in a matched case-control study, report that poor enhancement is more prevalent in patients with AT than in patients without AT, the clinical manifestations were not included in Lee’s matching criteria [24]. Using acute abdominal pain and pelvic mass as the inclusion criteria, we identified the ΔHU_PV-NC_ ≤ 17.5 HU as a CT sign with not only high sensitivity and uncompromised specificity but also the independent predictivity superior to all the other visually assessed CT signs, suggesting that this parameter might be the only CT sign to diagnose AT in patients with equivocal ultrasonographic impressions.

Color Doppler, when used to diagnose AT, has a sensitivity varying from 51% to 53% [6], so the color Doppler findings alone should not guide clinical decision-making by ruling out AT [26]. The different imaging mechanisms between color Doppler and contrast-enhance CT can explain why the ΔHU_PV-NC_ has superior sensitivity to the color Doppler findings. In the early phase of AT, the venous and lymphatic drainage is usually first impaired by the twisted vascular pedicle, leading to increased ovarian capillary hydrostatic pressure and tissue edema [27]. Given the quick distribution of the radiocontrast to the capillary bed and interstitial space, the reduced tissue perfusion caused by the elevated capillary hydrostatic pressure and edema can be characterized by the aberrantly changed CT numbers at a microcirculatory level [11, 12, 28]. Unlike contrast-enhanced CT, color Doppler imaging is based on the blood velocity referring to the large blood vessels that may still have blood flow during the early phase of AT [12, 29, 30]. Superior to the color Doppler examining the blood flow in a single phase, the ΔHU_PV-NC_ is generated in a “self-control” system within which the attenuations in both non-contrast and portal-venous phases were measured from the same patient. In the torsion vs non-torsion group, the opposite attenuation makes the ΔHU_PV-NC_ a highly sensitive and specific CT sign to differentiate twisted vs untwisted lesions. As a comparison, we have also assessed the attenuation difference between the non-contrast and equilibrium phases (ΔHU_EP-NC_). The sensitivity and specificity of ΔHU_EP-NC_ were lower than those of ΔHU_PV-NC_ (data not shown). The suboptimal performance of ΔHU_EP-NC_ suggests that the CT number of the images acquired at a late time point (equilibrium phase) post-contrast injection may have higher variabilities owing to the aberrant time-attenuation curve of the ischemic tissue, characterized by reduced peak attenuation, prolonged mean transit time, and delayed time to peak [31, 32].

Using contrast-enhanced ultrasonography to diagnose AT has been documented in case reports and case series studies [33, 34]. While the overall diagnostic accuracy reached 95% in the case series study, the small sample size of the control group (*n* = 2) makes this result less convincing [33]. A clinical trial has been initiated to assess the diagnostic accuracy of this approach [35]. Compared to ultrasonographic findings highly depending on the reader’s experience [36], the quantitative measurement-based ΔHU_PV-NC_ showed substantial post-training inter-rater agreement and increased diagnostic accuracy in the medical residents with two years of experience. With superior reproducibility among the less-experienced raters, the ΔHU_PV-NC_ should be a feasible CT sign adopted by properly trained junior physicians to improve their decision-making in an emergency setting.

Dual-energy CT acquires images at two energy levels simultaneously. By generating the tissue iodine concentration, the dual-energy CT demonstrates highly favorable sensitivity and specificity in distinguishing normal, ischemic, and infarcted myocardium [37]. Unlike heart infarction with arteriole end occlusions, the blood flow obstruction of AT starts from the venous end. Therefore, the dual-energy CT and tissue iodine concentration may provide new insights into the microcirculatory characteristics of twisted organs.

This retrospective study has limitations. First, many patients with painful pelvic mass were not given the contrast-enhanced CT as the CT is not considered a highly recommended imaging approach for the suspected patients [1, 38]. In addition to causing the small sample size in both derivation and mixed validation sets, the sporadically conducted enhanced CT scanning, particularly in patients without AT, may also bias the distribution of each non-torsion clinical condition in the control group, which could affect the specificity of the ΔHU_PV-NC_. Although the number of pooled patients (*n* = 218) with either ultrasonography-specified or ultrasonography-unspecified AT meets the minimal required sample size to estimate reliable sensitivity and specificity [39], the results of this study should be further verified in prospective studies with larger sample sizes. Second, the patient’s ultrasonographic results were not reevaluated in this study due to the lack of video records. Third, plotting the ROI requires experience, especially in lesions with limited parenchyma and thin lesion walls. Less-experienced readers need to be trained appropriately to reach a favorable diagnostic accuracy. Fourth, owing to the retrospective nature of this study, inconsistent image-acquiring conditions may increase the variabilities of ΔHU_PV-NC_, evidenced by the larger standard deviation of ΔHU_PV-NC_ in the non-torsion group than that in the torsion group. The different standard deviations between the two groups suggest that occluded blood perfusion in twisted organs reduces the CT number variabilities caused by inconsistent image-acquiring conditions. Therefore, the image acquisition-related inconsistency mainly impacts the specificity of the CT sign, especially with a cutoff value of 17.5 HU.

In conclusion, the introduction of contrast-enhanced CT can improve the diagnostic accuracy of AT in patients with equivocal ultrasonographic impressions. The added value of contrast-enhanced CT to diagnose AT after an uncertain ultrasonographic examination should be further evaluated in well-designed prospective studies.

## Supplementary information

ELECTRONIC SUPPLEMENTARY MATERIAL

## Data Availability

The data that support the findings of this study are available from the corresponding author Dr. Fuqing Zhou, upon reasonable request.

## Abbreviations

ΔHU_EP-NC_: Hounsfield unit difference between non-contrast and equilibrium phases
ΔHU_PV-NC_: Hounsfield unit difference between non-contrast and portal-venous phases
AT: Adnexal torsion
AUC: Area under the curve
HU: Hounsfield unit
ROC: Receiver operating characteristic curve
ROI: Region of interest
