# Supplementary material for: Decreased attenuation difference between non-contrast and portal-venous phases of CT predicts the ultrasonography-unspecified adnexal torsion
Source: Insights Imaging. 2025 Jan 10;16:12. doi: 10.1186/s13244-024-01885-4 (PMC11723866; doi:10.1186/s13244-024-01885-4)
Supplement: Supplementary file 1 — ELECTRONIC SUPPLEMENTARY MATERIAL [file 13244_2024_1885_MOESM1_ESM.pdf]

Decreased attenuation difference between non-contrast and portal-venous phases of CT predicts the ultrasonography-unspecified adnexal torsion

ELECTRONIC SUPPLEMENTARY MATERIAL

Table S1: Definition of visually assessed CT signs of adnexal torsion

CT Signs	Definition
Whirl sign	Twisted pedicle-like structure near the lesion.
Peritoneal ascites	Fluid collected in cul-de-sac.
Lesion wall thickening	Symmetrically or asymmetrically increased lesion wall thickness > 3 mm
Intralesional hemorrhage	Lesion components with the non-contrast attenuation > 50 HU without further increase in portal venous phase.
Fat stranding	Hazy increased attenuation of fat tissue around the lesion

Table S2: Patient's characteristics in derivation and validation sets

	Derivation			Validation		
	AT [3] (n = 47)	Non-AT (n = 58)	P [4] value	AT [3] (n = 26)	Non-AT (n = 34)	P [4] value
Age (y) [1]	44 ± 18	38 ± 14	0.09 [5]	53 ± 20	44 ± 116	0.053 [5]
Pain duration (d) [1]	5 ± 6	11 ± 9	0.001 [6]	6 ± 6	11 ± 10	0.03 [6]
Nausea or vomiting [2]	9 (19)	7 (12)	0.08	3 (12)	2 (6)	0.64
Neutrophil lymphocyte ratio	6.3 ± 4.6	4.7 ± 4.1	0.03 [6]	7.9 ± 7.8	4 ± 3.3	0.008 [6]
Largest lesion diameter (cm) [1]	11.6 ± 4.6	11 ± 6.1	0.026 [6]	11.2 ± 4.3	11.5 ± 6.3	0.87 [6]
Interval between CT and surgery (d)	3 ± 2	3 ± 2	0.08 [6]	4 ± 4	3 ± 2	0.9 [6]
Pathology [2]:						
Ovarian Cyst	15 (32)	15 (26)	0.52	8 (31)	7 (21)	0.39
Serous cystadenoma	9 (19)	5 (9)	0.15	4 (15)	2 (6)	0.38
Mucinous cystadenoma	5 (11)	8 (14)	0.77	4 (15)	5 (15)	0.1
Seromucinous cystadenoma	1 (2)	0 (0)	0.45	0 (0)	0 (0)	NA
Teratoma	9 (19)	5 (9)	0.15	2 (8)	5 (15)	0.69
Fibrothecoma	2 (4)	1 (2)	0.59	1 (4)	0 (0)	0.43
Endometrioma	1 (2)	10 (17)	0.02	1 (4)	4 (12)	0.38
Fallopian tube	2 (4)	0 (0)	0.2	1 (4)	0 (0)	0.43
Polycystic ovary syndrome	1 (2)	0 (0)	0.45	0 (0)	0 (0)	NA
Tubo-ovarian abscess	1 (2)	11 (19)	0.01	0 (0)	7 (21)	0.02
Fibroma	0 (0)	0 (0)	NA	1 (4)	0 (0)	0.43
Dysgerminoma	0 (0)	0 (0)	NA	1 (4)	0 (0)	0.43
Thecoma	0 (0)	0 (0)	NA	1 (4)	1 (3)	1
Paramesonephric duct cyst	0 (0)	0 (0)	NA	1 (4)	0 (0)	0.43
Mucinous cystadenocarcinoma	0 (0)	1 (2)	1	1 (4)	0 (0)	0.43
Borderline mucinous cystadenocarcinoma	0 (0)	1 (2)	1	0 (0)	2 (6)	0.5
Cortical inclusion cyst	1 (2)	0 (0)	0.45	0 (0)	0 (0)	NA
Serous cystadenocarcinoma	0 (0)	1 (2)	1	0 (0)	0 (0)	NA
Squamous cell carcinoma	0 (0)	0 (0)	NA	0 (0)	1 (3)	1

Note: NA-Not applicable.

1. Data are presented as mean ± SD.
2. Data are presented as the number of occurrences followed by (percentage).
3. AT: Adnexal torsion.
4. *P* value from Fisher's exact test except where otherwise indicated.
5. Student t-test.
6. Mann-Whitney U test.

Table S3 to S4 The sensitivity and specificity comparisons of $\Delta HU_{PV\text{-}NC} \leq 17.5$ HU between derivation and mixed validation sets (Fisher exact test).

Table S3: Comparison of the sensitivity of $\Delta HU_{PV\text{-}NC} \leq 17.5$ HU between derivation and mixed validation sets (Fisher exact test).

	$\Delta HU_{PV\text{-}NC} \leq 17.5$	$\Delta HU_{PV\text{-}NC} > 17.5$
Derivation	45	2
Mixed validation	24	2

P = 0.61

Table S4: Comparison of the specificity of $\Delta HU_{PV\text{-}NC} \leq 17.5$ HU between derivation and mixed validation sets (Fisher exact test).

	$\Delta HU_{PV\text{-}NC} \leq 17.5$	$\Delta HU_{PV\text{-}NC} > 17.5$
Derivation	7	51
Mixed validation	4	30

P = 1

Table S5 to S9: Sensitivity comparison (McNemar test) between the $\Delta HU_{PV\text{-}NC} \leq 17.5$ HU and visually assessed CT signs including the whirlpool sign, peritoneal ascites, lesion wall thickening, intralesional hemorrhage, and fat stranding.

Table S5: Sensitivity comparison (McNemar test) between the $\Delta HU_{PV\text{-}NC} \leq 17.5$ HU and whirlpool sign

	Whirlpool sign **+**	Whirlpool sign -
$\Delta HU_{PV\text{-}NC} \leq 17.5$	27	42
$\Delta HU_{PV\text{-}NC} > 17.5$	0	4

Chi-square: 42; $P < 0.001$

Table S6: Sensitivity comparison (McNemar test) between the $\Delta HU_{PV\text{-}NC} \leq 17.5$ HU and peritoneal ascites

	Peritoneal ascites **+**	Peritoneal ascites -
$\Delta HU_{PV\text{-}NC} \leq 17.5$	29	40
$\Delta HU_{PV\text{-}NC} > 17.5$	2	2

Chi-square: 34; $P < 0.001$

Table S7: Sensitivity comparison (McNemar test) between the $\Delta HU_{PV\text{-}NC} \leq 17.5$ HU and lesion wall thickening

	Wall thickening **+**	Wall thickening -
$\Delta HU_{PV\text{-}NC} \leq 17.5$	30	39
$\Delta HU_{PV\text{-}NC} > 17.5$	2	2

Chi-square: 33; $P < 0.001$

Table S8: Sensitivity comparison (McNemar test) between the $\Delta HU_{PV\text{-}NC} \leq 17.5$ HU and intralesional hemorrhage

	Hemorrhage **+**	Hemorrhage -
$\Delta HU_{PV\text{-}NC} \leq 17.5$	28	41
$\Delta HU_{PV\text{-}NC} > 17.5$	1	3

Chi-square: 38; $P < 0.001$

Table S9: Sensitivity comparison (McNemar test) between the $\Delta HU_{PV\text{-}NC} \leq 17.5$ HU and fat stranding

	Fat stranding **+**	Fat stranding -
$\Delta HU_{PV\text{-}NC} \leq 17.5$	30	39
$\Delta HU_{PV\text{-}NC} > 17.5$	2	2

Chi-square: 33; $P < 0.001$

Table S10 to S14: Specificity comparison (McNemar test) between the $\Delta HU_{PV\text{-}NC} \leq 17.5$ HU and visually assessed CT signs including the whirlpool sign, peritoneal ascites, lesion wall thickening, intralesional hemorrhage, and fat stranding.

Table S10: Specificity comparison (McNemar test) between the $\Delta HU_{PV\text{-}NC} \leq 17.5$ HU and Whirlpool sign

	Whirlpool sign **+**	Whirlpool sign -
$\Delta HU_{PV\text{-}NC} \leq 17.5$	2	9
$\Delta HU_{PV\text{-}NC} > 17.5$	4	77

Chi-square: 1.9; $P = 0.17$

Table S11: Specificity comparison (McNemar test) between the $\Delta HU_{PV\text{-}NC} \leq 17.5$ HU and peritoneal ascites

	Peritoneal ascites **+**	Peritoneal ascites -
$\Delta HU_{PV\text{-}NC} \leq 17.5$	2	9
$\Delta HU_{PV\text{-}NC} > 17.5$	25	56

Chi-square: 7.5; $P = 0.006$

Table S12: Specificity comparison (McNemar test) between the $\Delta HU_{PV\text{-}NC} \leq 17.5$ HU and lesion wall thickening

	Wall thickening **+**	Wall thickening -
$\Delta HU_{PV\text{-}NC} \leq 17.5$	3	8
$\Delta HU_{PV\text{-}NC} > 17.5$	11	70

Chi-square: 0.47; $P = 0.49$

Table S13: Specificity comparison (McNemar test) between the $\Delta HU_{PV\text{-}NC} \leq 17.5$ HU and intralesional hemorrhage

	Hemorrhage **+**	Hemorrhage -
$\Delta HU_{PV\text{-}NC} \leq 17.5$	3	9
$\Delta HU_{PV\text{-}NC} > 17.5$	19	62

Chi-square: 3.6; $P = 0.06$

Table S14: Specificity comparison (McNemar test) between the $\Delta HU_{PV\text{-}NC} \leq 17.5$ HU and fat stranding

	Fat stranding **+**	Fat stranding -
$\Delta HU_{PV\text{-}NC} \leq 17.5$	3	8
$\Delta HU_{PV\text{-}NC} > 17.5$	11	70

Chi-square: 18.8; $P < 0.001$

Table S15: The pathological characteristics of the patients who were either false positive or false negative for the CT sign of $\Delta HU_{PV-NC} \leq 17.5$ HU.

Torsion (n = 4; $\Delta HU_{PV-NC} > 17.5$ HU; false negative)	Non-Torsion (n = 11; $\Delta HU_{PV-NC} \leq 17.5$ HU; false positive)
Mucinous cystadenocarcinoma (n = 1)	Mucinous cystadenoma (n = 3)
Dysgerminoma (n = 1)	Teratoma (n = 3)
Endometrioma (n = 1)	Ovarian cyst (n = 2)
Tubo-ovarian abscess (n = 1)	Fibrothecoma (n = 1)
	Endometrioma (n = 1)
	Serous cystadenoma (n = 1)

Table S16: Procedure of backward elimination of the multivariable logistic regression to predict adnexal torsion

Step	Removed predictor	Remaining predictor	Wald test	P value	Odds Ratio (95% CI)
1	No	$\Delta HU_{PV\text{-}NC} \leq 17.5$	50.9	< 0.001	111.8 (30.6, 407.9)
		Fat stranding	1.98	0.16	2.6 (0.7, 9.7)
		Lesion wall thickening	1.74	0.19	2.5 (0.6, 9.5)
		Intralesional hemorrhage	0.9	0.34	1.9 (0.5, 6.8)
		Whirlpool sign	0.24	0.63	1.5 (0.3, 6.6)
2	Whirlpool sign	$\Delta HU_{PV\text{-}NC} \leq 17.5$	55.5	< 0.001	121.8 (34.4, 431.2)
		Fat stranding	2.3	0.13	2.8 (0.8, 10.1)
		Lesion wall thickening	2.4	0.12	2.7 (0.8, 9.9)
		Intralesional hemorrhage	0.8	0.37	1.8 (0.5, 6.6)
3	Intralesional hemorrhage	$\Delta HU_{PV\text{-}NC} \leq 17.5$	56	< 0.001	125.5 (35.4, 445.1)
		Fat stranding	3.1	0.08	3.1 (0.9, 10.9)
		Lesion wall thickening	2.4	0.12	2.7 (0.8, 9.3)
4	Lesion wall thickening	$\Delta HU_{PV\text{-}NC} \leq 17.5$	59.3	< 0.001	137 (39, 481)
		Fat stranding	3.6	0.057	3.4 (1, 11.8)

Table S17: Diagnostic performance and logistic regression of CT signs to predict adnexal torsion in patients with confident ultrasonographic impression.

CT Signs	Sn [2] (%)	*P* Value [4]	Sp [3] (%)	*P* Value [4]	Diagnostic Accuracy	*P* Value [4]	LR+ [5]	LR- [6]	Univariable regression	
									OR [7]	*P* value
$\Delta HU_{PV-NC} \leq$ 17.5 HU [1]	95 (82, 99)	NA [8]	81 (54, 96)	NA [8]	88 (75, 96)	NA[8]	5.4 (1.8, 14)	0.07 (0.02, 0.26)	76 (11, 506)	<0.001
Whirlpool sign	86 (71, 95)	0.45	25 (7, 52)	0.01	68 (54, 80)	0.013	1.2 (0.85, 1.57)	0.5 (0.17, 1.75)	2.1 (0.5, 9.3)	0.313
Fat stranding	54 (37, 71)	<0.001	63 (35, 85)	0.5	57 (42, 70)	<0.001	1.4 (0.72, 2.9)	0.7 (0.44, 1.23)	2.0 (0.6, 6.5)	0.272
Peritoneal ascites	51 (34, 68)	0.001	56 (30, 80)	0.5	53 (39, 67)	<0.001	1.2 (0.62, 2.22)	0.86 (0.5, 1.49)	1.4 (0.4, 4.4)	0.612
Lesion wall thickening	81 (65, 92)	0.06	31 (11, 59)	0.008	66 (52, 78)	<0.001	1.2 (0.82, 1.7)	0.6 (0.23, 1.62)	1.9 (0.5, 7.4)	0.329
Intralesional hemorrhage	46 (29, 63)	<0.001	69 (41, 89)	0.5	53 (39, 67)	<0.001	1.5 (0.66, 3.29)	0.8 (0.5, 1.23)	1.9 (0.5, 6.5)	0.322

Note: Values in parentheses are 95% CIs.

1. ΔHU_{PV-NC}: The Hounsfield unit difference between non-contrast and portal-venous phases.
2. Sn: Sensitivity
3. Sp: Specificity
4. *P* value of the comparison between visually assessed CT signs and $\Delta HU_{PV-NC} \leq$ 17.5 HU (McNemar Test).
5. LR+: Positive likelihood ratio.
6. LR-: Negative likelihood ratio.
7. OR: Diagnostic odds ratio.
8. NA: Not applicable.

Table S18 to S19 The sensitivity and specificity comparisons of $\Delta HU_{PV-NC} \leq 17.5$ HU between ultrasound-unspecified patients and pooled patients with either ultrasound-unspecified or ultrasound-specified adnexal torsion (Fisher exact test).

Table S18: Comparison of the sensitivity of $\Delta HU_{PV-NC} \leq 17.5$ HU

	$\Delta HU_{PV-NC} \leq 17.5$	$\Delta HU_{PV-NC} > 17.5$
Ultrasound-unspecified	69	4
Ultrasound-unspecified plus Ultrasound-specified	104	6

P = 1

Table S19: Comparison of the specificity of $\Delta HU_{PV-NC} \leq 17.5$ HU

	$\Delta HU_{PV-NC} \leq 17.5$	$\Delta HU_{PV-NC} > 17.5$
Ultrasound-unspecified	11	81
Ultrasound-unspecified plus Ultrasound-specified	14	94

P = 1

Table S20 to S23: Inter-rater agreement between the consensus and each resident before and after the training with $\Delta HU_{PV\text{-}NC}$ measurement and calculation.

Table S20: Pre-training agreement between resident-1 and consensus

	Pre-training Yes	Pre-training No
Consensus Yes	82	4
Consensus No	53	26

Cohen's kappa: 0.29 (95% CI: 0.17, 0.41)

Table S21: Pre-training agreement between resident-2 and consensus

	Pre-training Yes	Pre-training No
Consensus Yes	64	21
Consensus No	41	39

Cohen's kappa: 0.24 (95% CI: 0.1, 0.39)

Table S22: Post-training agreement between resident-1 and consensus

	Pre-training Yes	Pre-training No
Consensus Yes	74	11
Consensus No	10	70

Cohen's kappa: 0.75 (95% CI: 0.65, 0.85)

Table S23: Post-training agreement between resident-2 and consensus

	Pre-training Yes	Pre-training No
Consensus Yes	68	17
Consensus No	6	74

Cohen's kappa: 0.72 (95% CI: 0.62, 0.83)

Table S24 to S27: Diagnostic accuracy of two residents before and after being trained with the ΔHU_{PV-NC} measurement and calculation.

Table S24: Pre-training diagnostic accuracy of resident-1

	Torsion **+**	Torsion -
Yes	24	6
No	49	86

Diagnostic accuracy (%): 67 (95% CI: 59, 74).

Table S25: Pre-training diagnostic accuracy of resident-2

	Torsion **+**	Torsion -
Yes	38	21
No	35	71

Diagnostic accuracy (%): 66 (95% CI: 58, 73).

Table S26: Post-training diagnostic accuracy of resident-1

	Torsion **+**	Torsion -
Yes	61	19
No	12	73

Diagnostic accuracy (%): 81 (95% CI: 74, 87).

Table S27: Post-training diagnostic accuracy of resident-2

	Torsion **+**	Torsion -
Yes	66	25
No	7	67

Diagnostic accuracy (%): 81 (95% CI: 74, 87).

Table S28 to S29: Comparison of pre- vs post-training diagnostic accuracy of two residents with two years of experience.

Table S28: Comparison (McNemar test) of pre- vs post-training diagnostic accuracy of resident-1

	Pre-training Correct	Pre-training Wrong
Post-training Correct	87	45
Post-training Wrong	23	10

Chi-square: 7.1; *P* = 0.007

Table S29: Comparison (McNemar test) of pre- vs post-training diagnostic accuracy of resident-2

	Pre-training Correct	Pre-training Wrong
Post-training Correct	86	47
Post-training Wrong	22	10

Chi-square: 9.1; *P* = 0.002

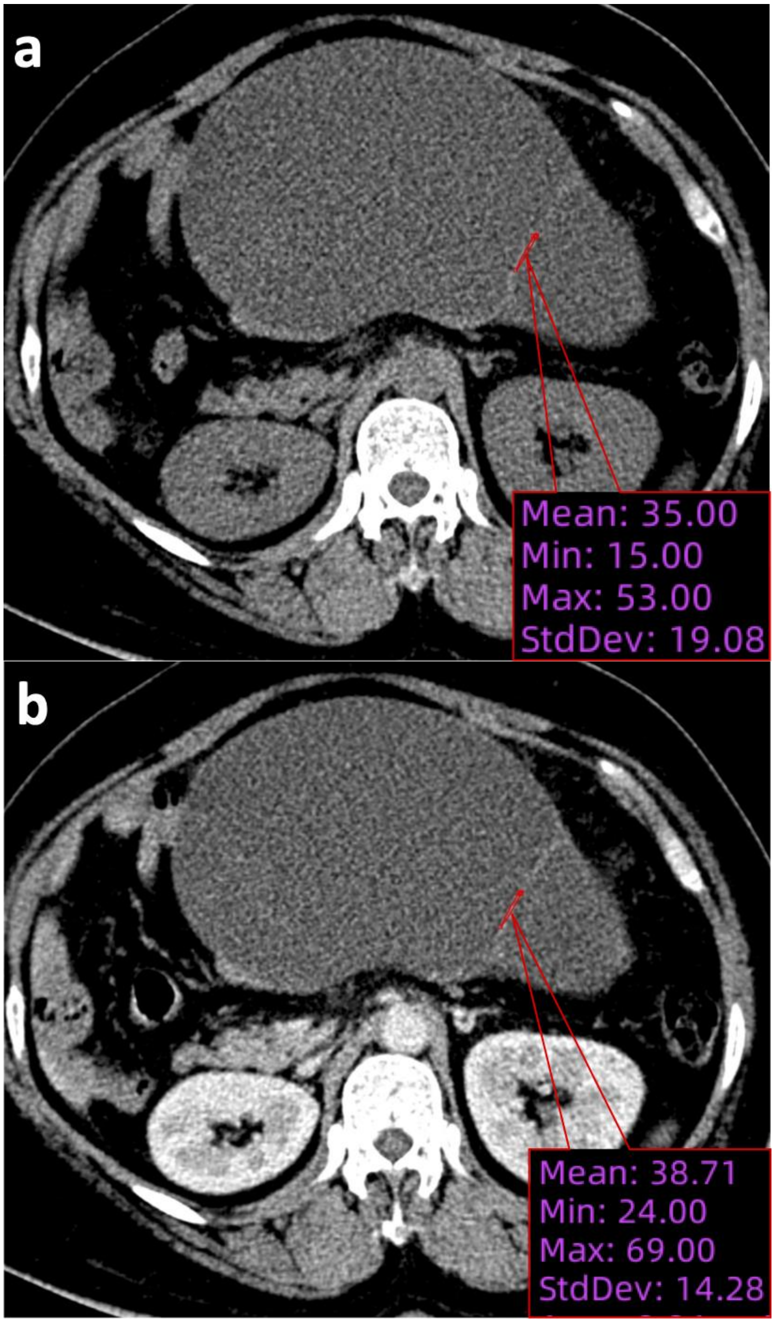

Figure S1: Attenuation measurement in contrast-enhanced CT image of a patient (age: 53 years) with a chief complaint of abdominal pain for 7 days. Surgically and pathologically confirmed non-twisted mucinous cystadenoma. (a) Non-contrast phase (attenuation: 35 HU). (b) Portal-venous phase (attenuation: 38.7 HU). The Hounsfield unit difference between non-contrast and portal-venous phases (ΔHU_{PV-NC}) is 3.7 HU.

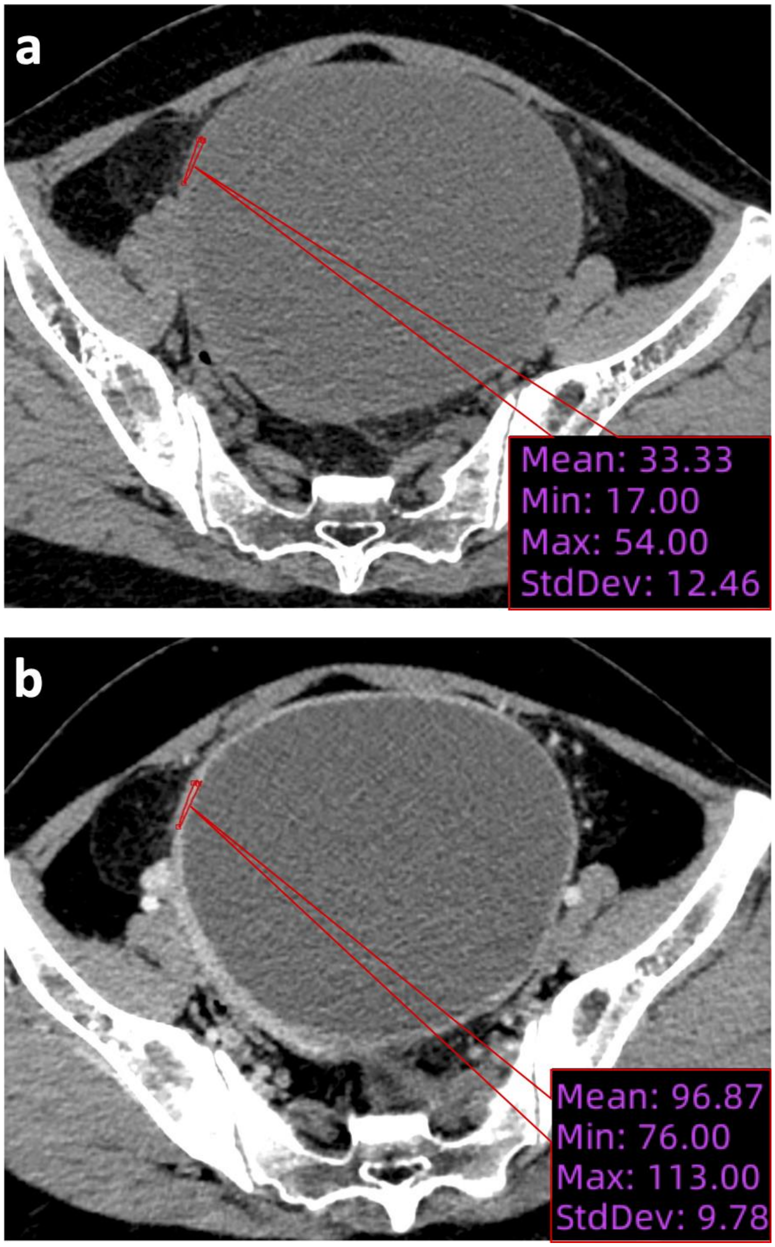

Figure S2: Attenuation measurement in contrast-enhanced CT image of a patient (age: 39 years) with a chief complaint of abdominal pain for 15 days. Surgically and pathologically confirmed twisted tubo-ovarian abscess. (a) Non-contrast phase (attenuation: 33.3 HU). (b) Portal-venous phase (attenuation: 96.9 HU). The Hounsfield unit difference between non-contrast and portal-venous phases (ΔHU_{PV-NC}) is 63.5 HU.

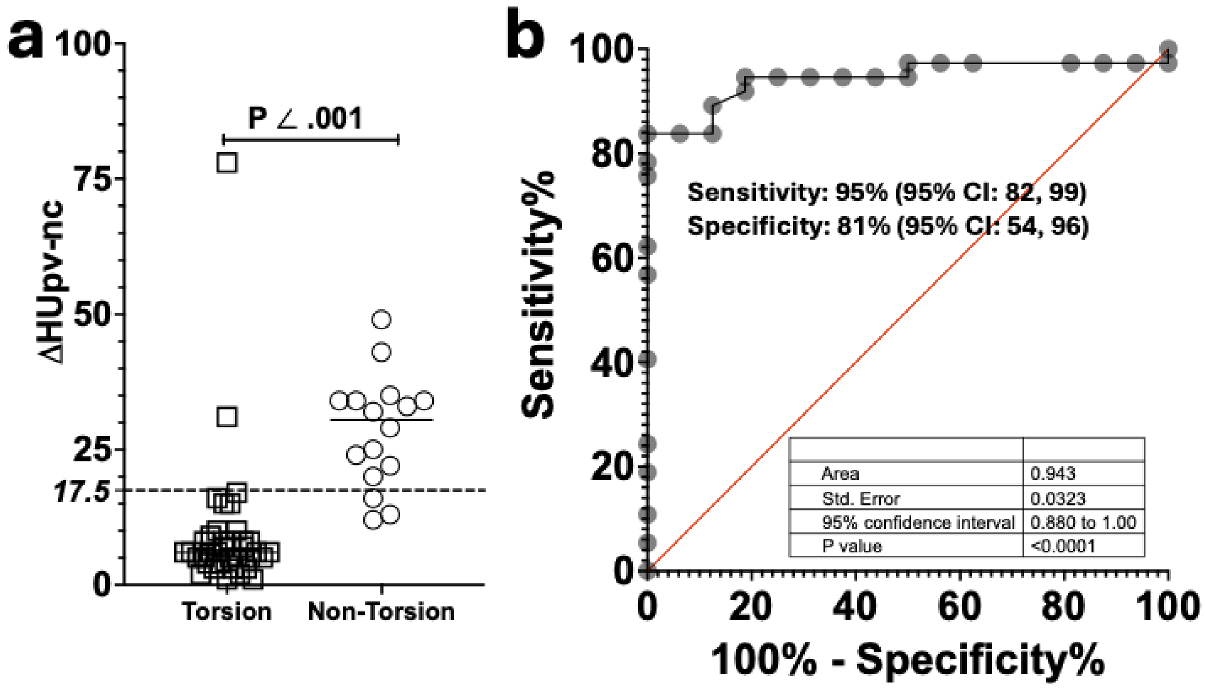

Figure S3: Hounsfield unit difference between non-contrast and portal-venous phases ($\Delta HU_{PV\text{-}NC}$) in patients with ultrasound suspicion of adnexal torsion (n = 53).
(a) $\Delta HU_{PV\text{-}NC}$ (cutoff value: 17.5 HU) of twisted (n =37) vs non-twisted (n = 16) lesions. Torsion: 9.3 ± 12.8 HU; non-torsion: 28.4 ± 10.4 HU ($P < 0.001$).
(b) The receiver operating characteristic curve (ROC) of the $\Delta HU_{PV\text{-}NC}$ (Torsion: n = 37; Non-torsion: n = 16). With the cutoff value of 17.5 HU, the sensitivity and specificity are 95% (95% CI: 82, 99) and 81% (95% CI: 54, 96).

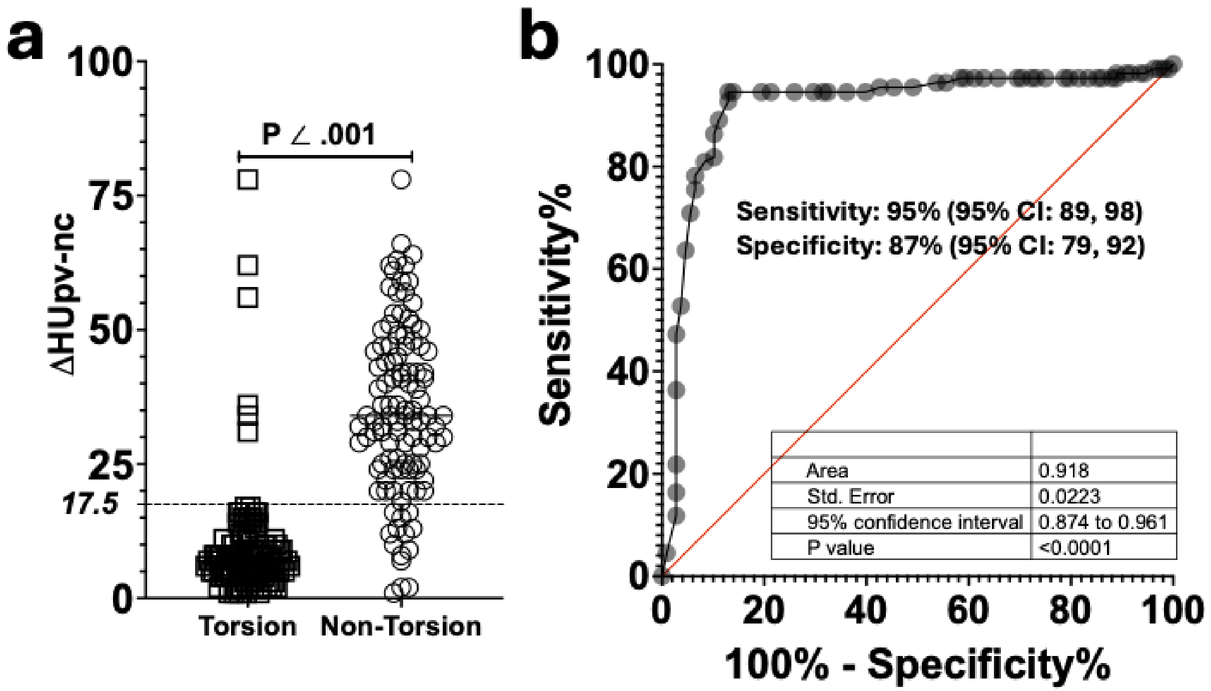

Figure S4: Hounsfield unit difference between non-contrast and portal-venous phases ($\Delta HU_{PV\text{-}NC}$) in pooled patients with either ultrasonography-specified or ultrasound–specified adnexal torsion (n = 218).

(a) $\Delta HU_{PV\text{-}NC}$ (cutoff value: 17.5 HU) of twisted (n = 110) vs non-twisted (n = 108) lesions. Torsion: 9.7 ± 11.2 HU; non-torsion: 34.9 ± 15.7 HU ($P < 0.001$).

(b) The receiver operating characteristic curve (ROC) of the $\Delta HU_{PV\text{-}NC}$ (Torsion: n = 110; Non-torsion: n = 108). With the cutoff value of 17.5 HU, the sensitivity and specificity are 95% (95% CI: 89, 98) and 87% (95% CI: 79, 92).
